# Supplementary figures and images for: Stable Host Gene Expression in the Gut of Adult Drosophila melanogaster with Different Bacterial Mono-Associations
Source: PLoS One. 2016 Nov 29;11(11):e0167357. doi: 10.1371/journal.pone.0167357 (PMC5127555; doi:10.1371/journal.pone.0167357)

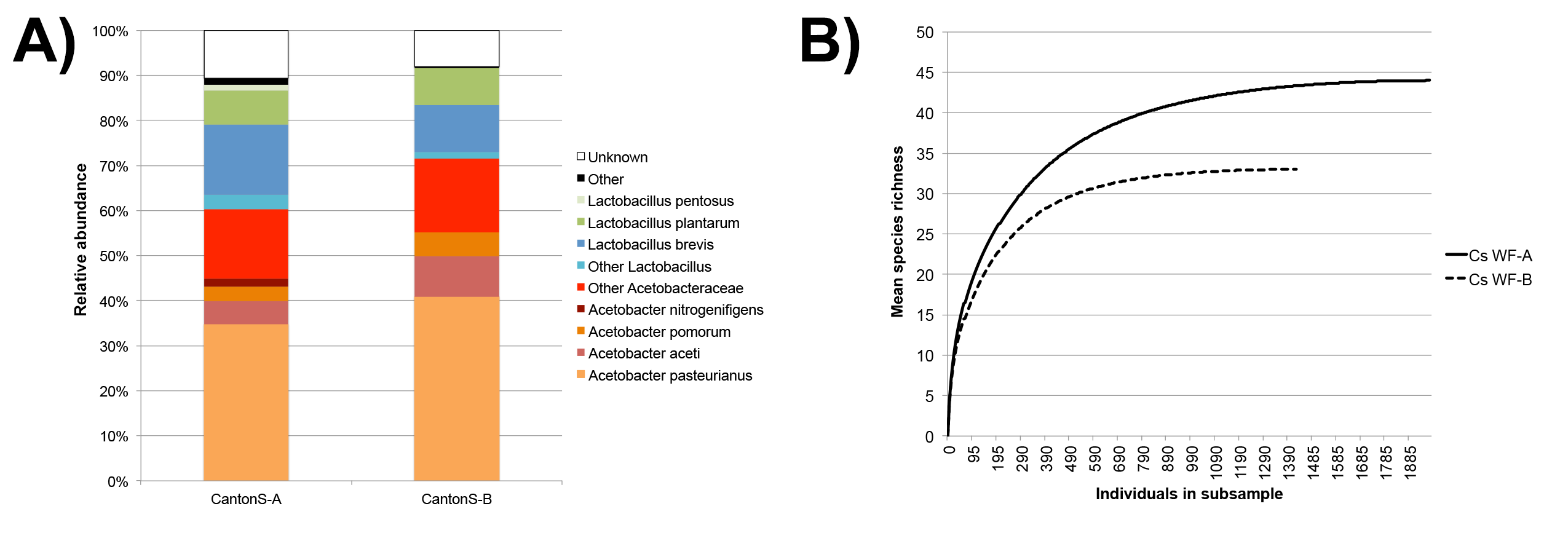

Supplement: S1 Fig — A) Relative species abundance of gut bacteria as determined by shotgun sequencing reads to the Green Genes 16S rRNA database release 13–5. “Other” category includes species not listed in key. “Unknown” category includes reads that aligned to 16S rRNA sequences included in the Green Genes database annotated as “unknown” (e.g. unknown compost). B) Rarefaction curve using data shown in A. (TIF) [file pone.0167357.s001.tif]

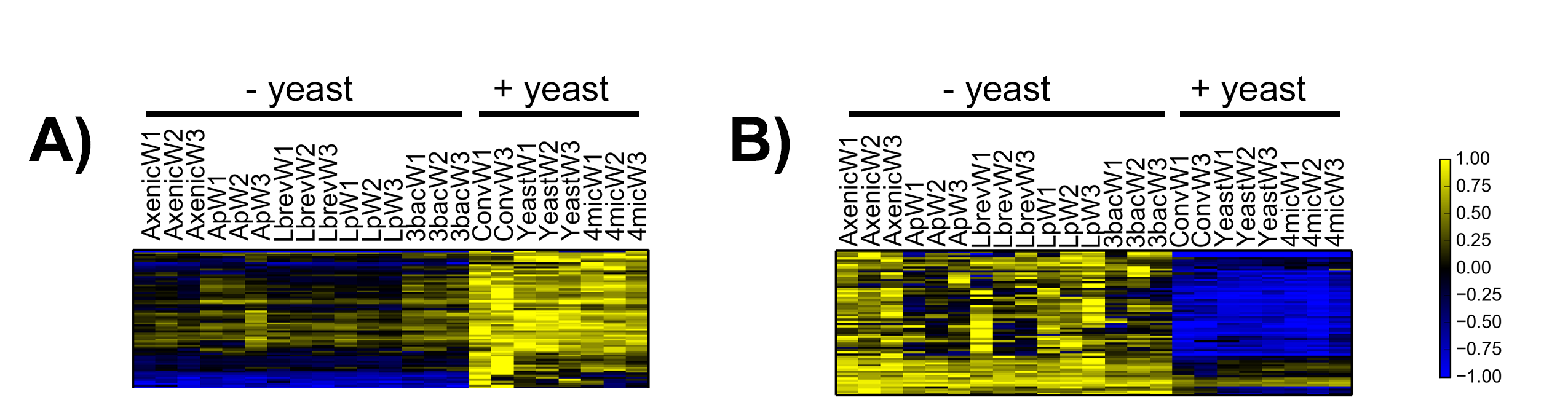

Supplement: S2 Fig — A) heatmap of 72 genes that are overexpressed in conventional, yeast- mono- and poly-associated whole flies compared to other whole fly samples (Bonferroni p-value>0.05, ANOVA). B) Heatmap of 67 genes that are overexpressed in axenic and bacteria mono- and poly-associated whole flies compare to other whole fly samples (Bonferroni p-value>0.05, ANOVA). Abbreviations: Ap = A. pasteurianus-mono-associated; Lbrev = L. brevis-mono-associated, Lp = L. plantarum-mono-associated, 3bac = poly-associated without yeast, Ax = axenic, Conv = conventional, Yeast = S. cerevisiae-mono-associated, 4mic = poly-associated with yeast. (TIF) [file pone.0167357.s002.tif]

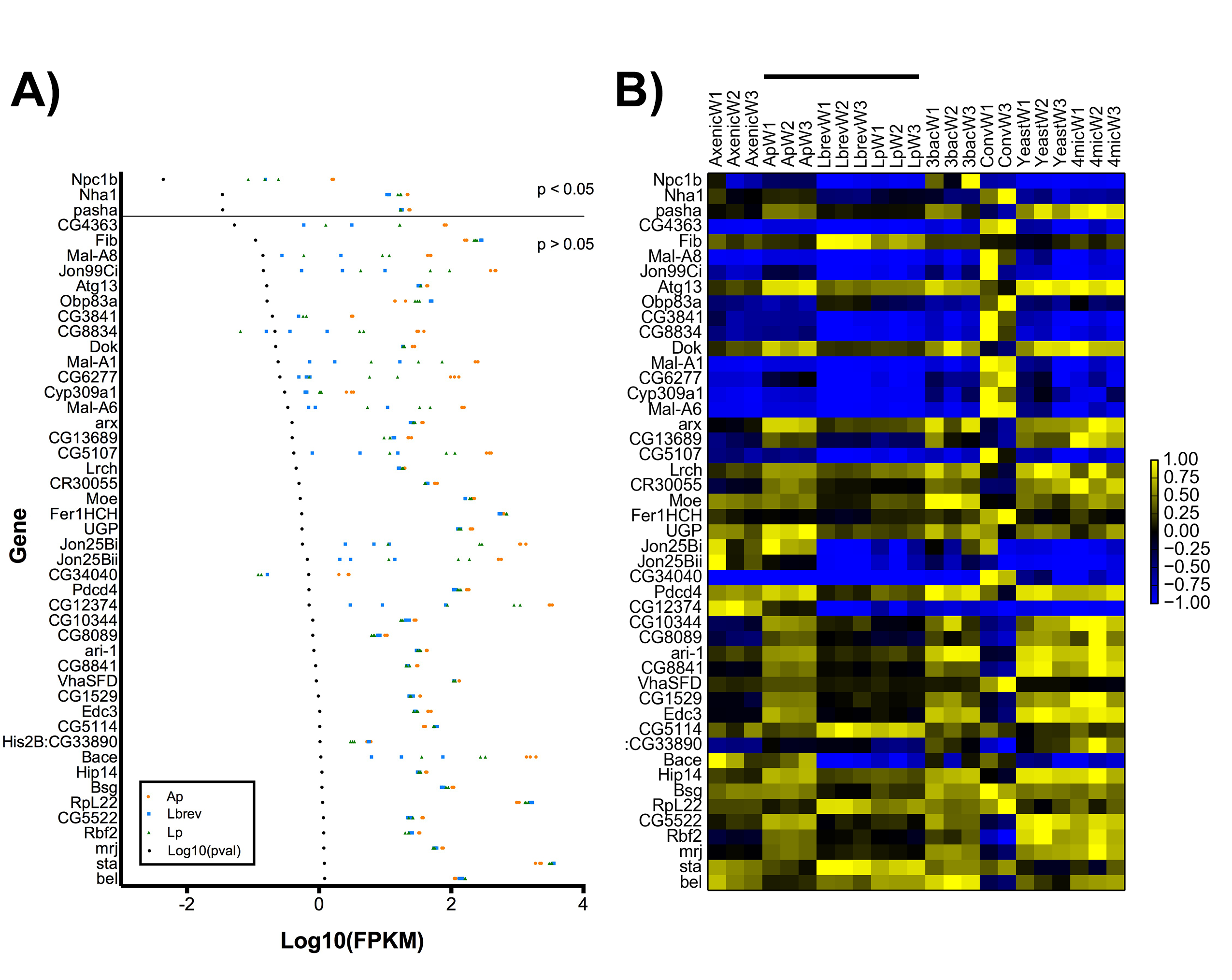

Supplement: S3 Fig — A) Scatterplot of log10-transformed FPKM values for each bacteria mono-associated whole fly replicate. Genes are ordered from lowest ANOVA p-value (top) to highest (bottom). P-values have undergone a Bonferroni correction for multiple testing. B) Data from A presented as a heatmap. FPKM values for each gene are normalized to range from -1 to 1 before plotting. Black line above heatmap denotes bacteria mono-association samples. Abbreviations: Ap = A. pasteurianus-mono-associated; Lbrev = L. brevis-mono-associated, Lp = L. plantarum-mono-associated, 3bac = poly-associated without yeast, Ax = axenic, Conv = conventional, Yeast = S. cerevisiae-mono-associated, 4mic = poly-associated with yeast. (TIF) [file pone.0167357.s003.tif]

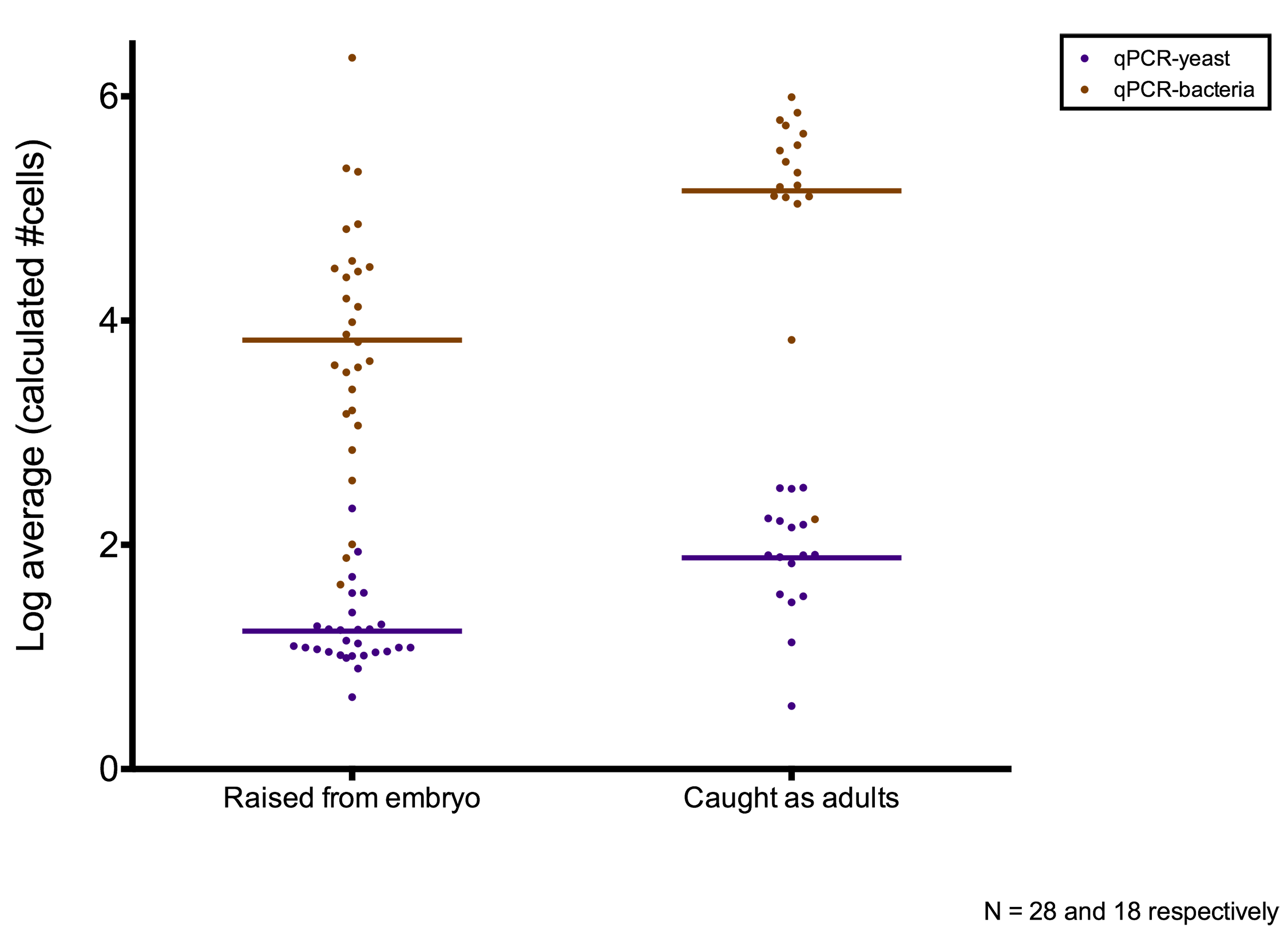

Supplement: S4 Fig — Log10 transformed average number of estimated bacteria cells or yeast cells by qPCR for individual, female, wild D. melanogaster raised from embryos (ranging from 3–10 days post-eclosion) or caught as adults (of unknown age). The mean for each group is plotted as a horizontal line. (TIF) [file pone.0167357.s004.tif]

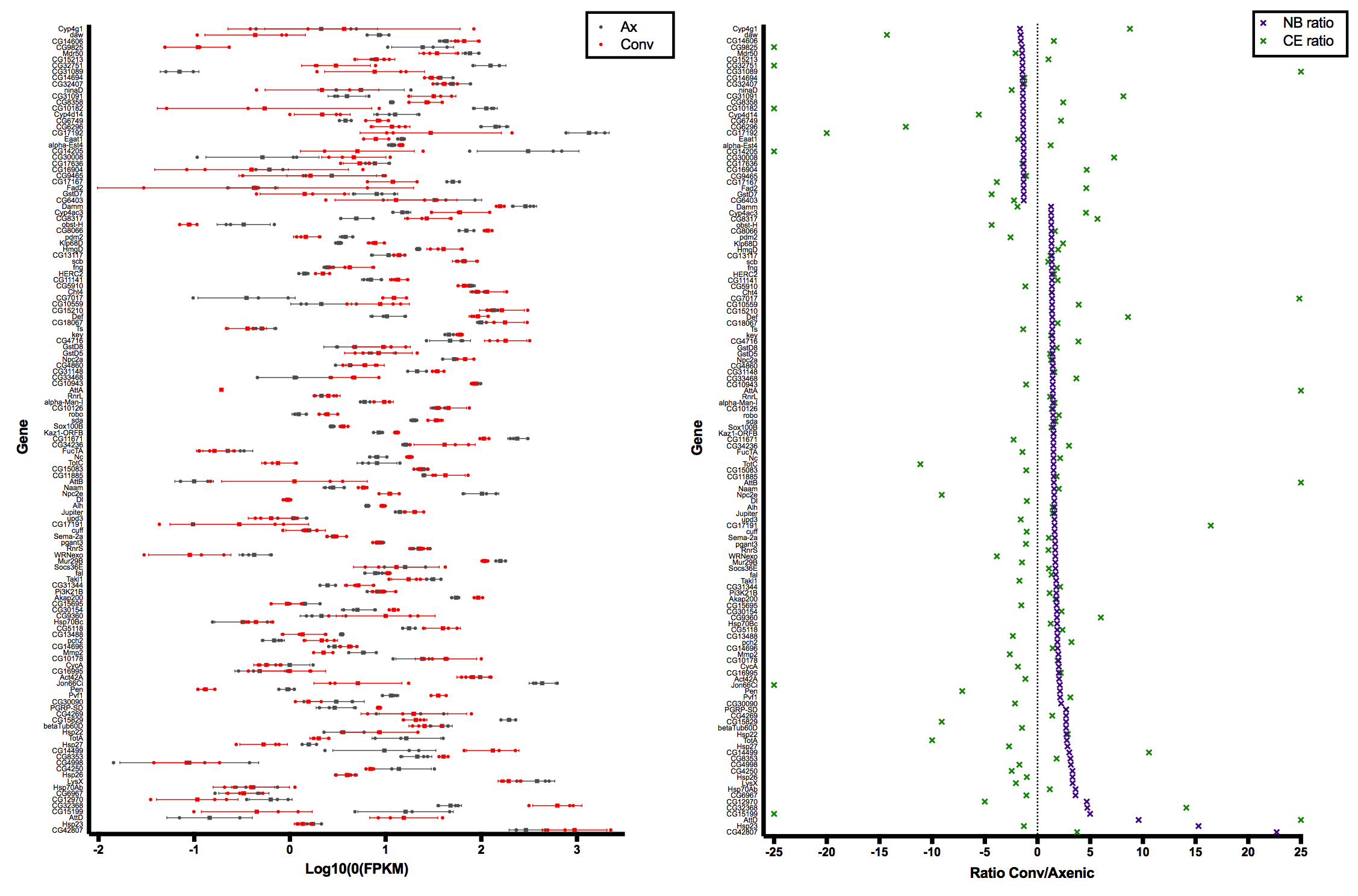

Supplement: S5 Fig — Left) Log10 of FPKM values for each of the genes in [23] core set in present manuscript’s gut RNAseq data. Individual replicates are shown as dots. Standard deviation is shown as error bars with mean bolded. Right) Ratio of expression in conventional over axenic treatments for each of the genes in the core set in [23] Affymetrix data and in present manuscript’s gut RNAseq data. For both plots, genes are ordered by the ratio of gene expression (conventional over axenic) as reported in [23]. For right plot, genes with a ratio lower than -25 have been plotted as exactly -25; genes with ratio higher than 25 have been plotted as exactly 25. NB ratio = data from Broderick et al, 2014; CE ratio = data from this study. (TIFF) [file pone.0167357.s005.tiff]

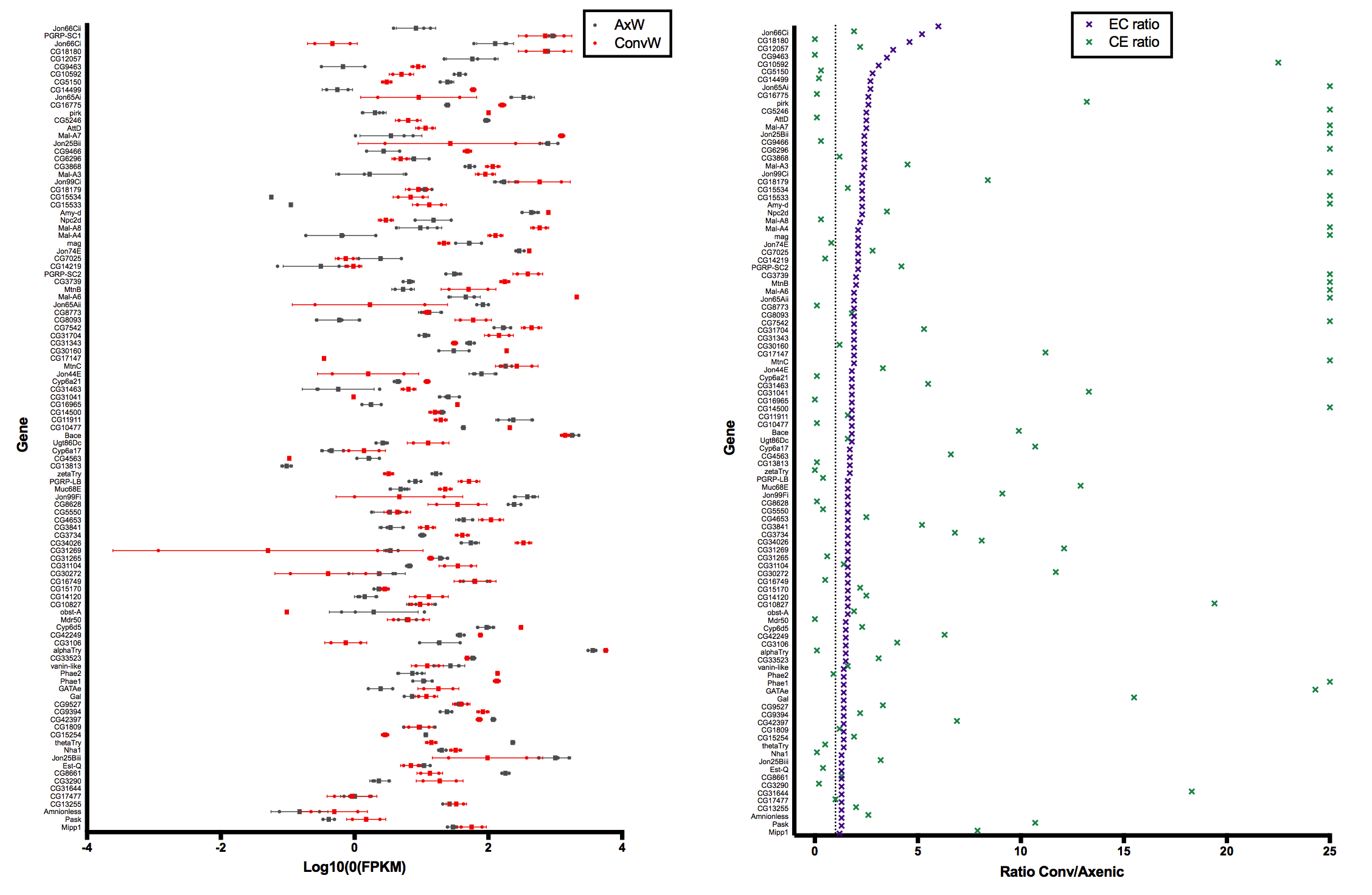

Supplement: S6 Fig — Left) Log10 of FPKM values for each of the genes in [11] in present manuscript’s whole fly RNAseq data. Individual replicates are shown as dots. Standard deviation is shown as error bars with mean bolded. Right) Ratio of expression in conventional over axenic treatments for each of the genes in the core set in [11] Affymetrix data and in present manuscript’s whole fly RNAseq data. For both plots, genes are ordered by the ratio of gene expression (conventional over axenic) as reported in [11]. For right plot, genes with ratio higher than 25 have been plotted as exactly 25. EB ratio = data from Erkosar Combe et al, 2014; CE ratio = data from this study. (TIFF) [file pone.0167357.s006.tiff]

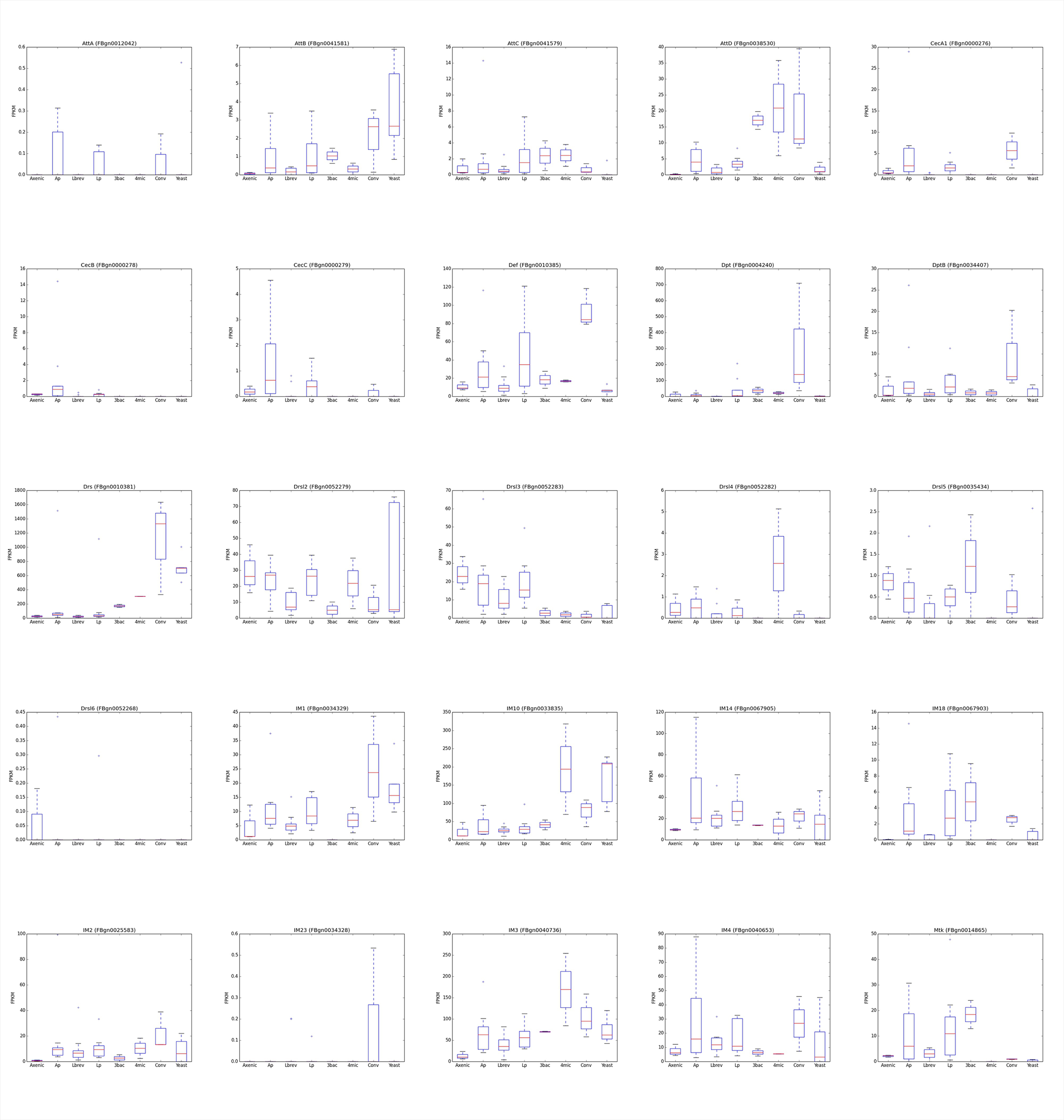

Supplement: S7 Fig — Data are plotted as boxes and whiskers using boxplot function in Matplotlib (Python). Each box is drawn from the lower to upper quartile values for all replicates for the indicated sample. The horizontal line in the box indicates the median. Whiskers extend 1.5 times the length of the box. Points beyond whiskers (outliers) are plotted as crosses. AMP genes for which FPKM values are zero across all treatments (i.e. are universally unexpressed) are omitted. (TIF) [file pone.0167357.s007.tif]

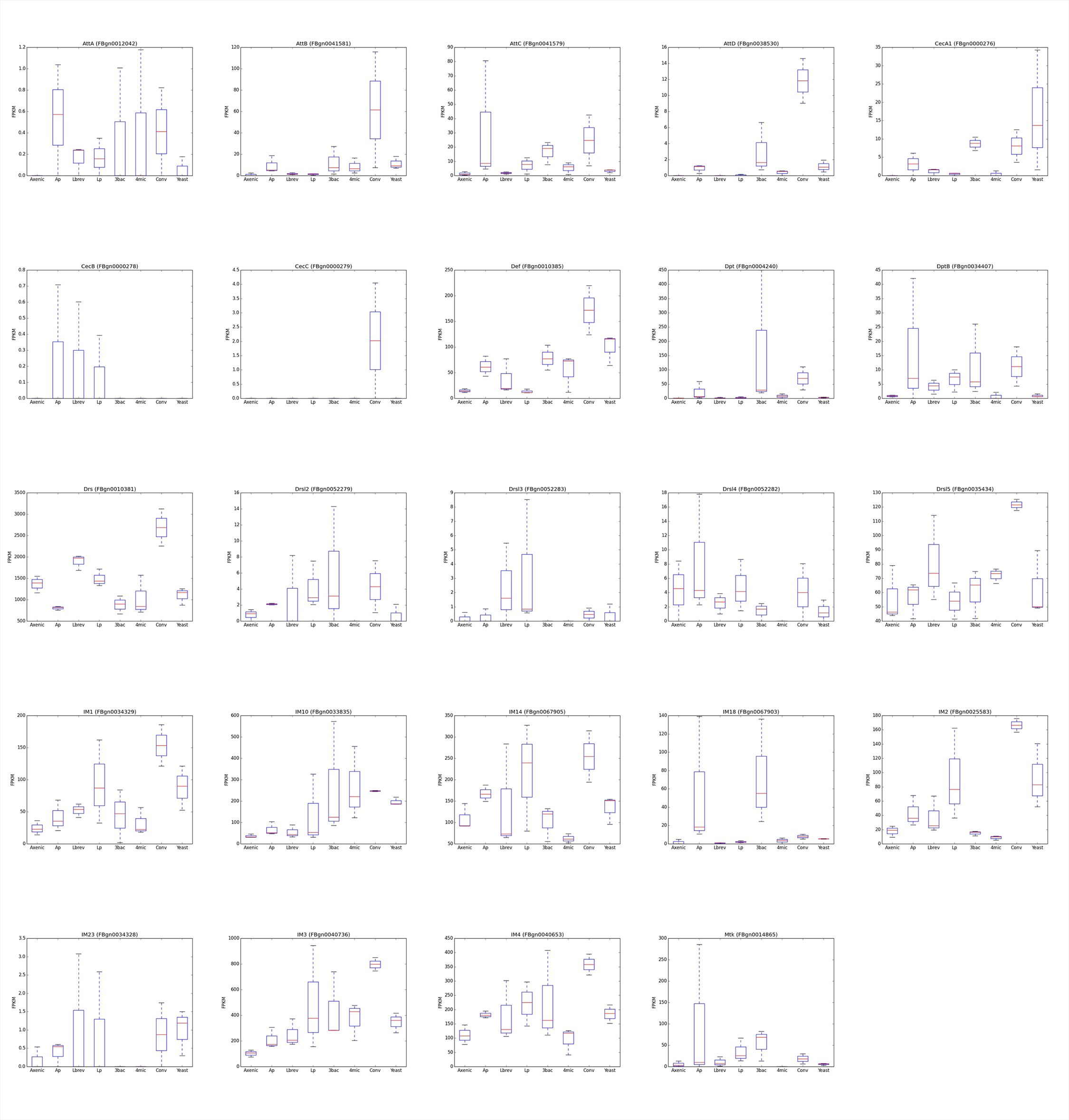

Supplement: S8 Fig — Data are plotted as boxes and whiskers using boxplot function in Matplotlib (Python). Each box is drawn from the lower to upper quartile values for all replicates for the indicated sample. The horizontal line in the box indicates the median. Whiskers extend 1.5 times the length of the box. Points beyond whiskers (outliers) are plotted as crosses. AMP genes for which FPKM values are zero across all treatments (i.e. are universally unexpressed) are omitted. (TIF) [file pone.0167357.s008.tif]
