# Supplementary material for: Stable Host Gene Expression in the Gut of Adult Drosophila melanogaster with Different Bacterial Mono-Associations
Source: PLoS One. 2016 Nov 29;11(11):e0167357. doi: 10.1371/journal.pone.0167357 (PMC5127555; doi:10.1371/journal.pone.0167357)
Supplement: S1 Table — (DOCX) [file pone.0167357.s009.docx]

| **Lane** | **Read type^** | **Sample type** | **Sample ID** | **Total input read pairs*** | **Concordantly-aligned pairs*** | **Concordant alignment rate*** |
| --- | --- | --- | --- | --- | --- | --- |
| 1 | 100 PE | Gut | Ap1a | 4.53E+07 | 2.84E+07 | 62.6 |
| 1 | 100 PE | Gut | Ap1b | 2.74E+07 | 1.70E+07 | 61.8 |
| 1 | 100 PE | Gut | Ap1c | 4.96E+07 | 3.10E+07 | 62.5 |
| 1 | 100 PE | Gut | Lbrev1a | 1.39E+07 | 8.61E+06 | 61.9 |
| 1 | 100 PE | Gut | Lbrev1b | 2.08E+07 | 1.30E+07 | 62.2 |
| 1 | 100 PE | Gut | Lbrev1c | 2.06E+07 | 1.22E+07 | 59.1 |
| 1 | 100 PE | Gut | Lp1a | 1.94E+07 | 1.17E+07 | 60.6 |
| 1 | 100 PE | Gut | Lp1b | 2.46E+07 | 1.51E+07 | 61.3 |
| 1 | 100 PE | Gut | Lp1c^x^ | 5.41E+07 | 3.53E+07 | 65.2 |
| 2 | 100 PE | Gut | Ap2a | 1.14E+07 | 8.56E+06 | 74.9 |
| 2 | 100 PE | Gut | Ap2b | 2.12E+07 | 1.63E+07 | 76.9 |
| 2 | 100 PE | Gut | Ap2c | 2.05E+07 | 1.57E+07 | 76.5 |
| 2 | 100 PE | Gut | Lbrev2a | 1.71E+07 | 1.28E+07 | 75.2 |
| 2 | 100 PE | Gut | Lbrev2b | 1.84E+07 | 1.39E+07 | 75.7 |
| 2 | 100 PE | Gut | Lbrev2c | 1.34E+07 | 1.05E+07 | 78.0 |
| 2 | 100 PE | Gut | Lp2a | 2.37E+07 | 1.80E+07 | 76.0 |
| 2 | 100 PE | Gut | Lp2b | 4.64E+07 | 3.55E+07 | 76.5 |
| 2 | 100 PE | Gut | Lp2c | 3.92E+07 | 3.01E+07 | 76.8 |
| 3 | 100 PE | Gut | Ap3a | 3.88E+07 | 3.06E+07 | 78.9 |
| 3 | 100 PE | Gut | Ap3b | 1.57E+07 | 1.22E+07 | 77.5 |
| 3 | 100 PE | Gut | Ap3c | 2.19E+07 | 1.70E+07 | 77.4 |
| 3 | 100 PE | Gut | Lbrev3a | 1.54E+07 | 1.20E+07 | 77.9 |
| 3 | 100 PE | Gut | Lbrev3b | 2.07E+07 | 1.60E+07 | 77.4 |
| 3 | 100 PE | Gut | Lbrev3c | 2.21E+07 | 1.71E+07 | 77.4 |
| 3 | 100 PE | Gut | Lp2c | 1.70E+07 | 1.34E+07 | 78.4 |
| 3 | 100 PE | Gut | Lp3a | 2.61E+07 | 2.00E+07 | 76.8 |
| 3 | 100 PE | Gut | Lp3b | 2.05E+07 | 1.57E+07 | 76.5 |
| 3 | 100 PE | Gut | Lp3c | 2.19E+07 | 1.68E+07 | 76.7 |
| 4 | 100 PE | Gut | Ax1 | 1.58E+07 | 1.19E+07 | 75.7 |
| 4 | 100 PE | Gut | Ax2 | 3.34E+07 | 2.56E+07 | 76.6 |
| 4 | 100 PE | Gut | Ax3 | 2.96E+07 | 2.28E+07 | 77.2 |
| 4 | 100 PE | Gut | Conv1 | 3.86E+07 | 2.98E+07 | 77.3 |
| 4 | 100 PE | Gut | Conv2 | 2.50E+07 | 1.92E+07 | 77.0 |
| 4 | 100 PE | Gut | Conv3 | 3.33E+07 | 2.54E+07 | 76.4 |
| 4 | 100 PE | Gut | Yeast1^x^ | 3.26E+07 | 2.67E+07 | 81.8 |
| 4 | 100 PE | Gut | Yeast2 | 1.65E+07 | 1.25E+07 | 75.7 |
| 4 | 100 PE | Gut | Yeast3 | 2.10E+07 | 1.59E+07 | 75.5 |
| 5 | 100 PE | Whole | ApW1 | 6.75E+06 | 5.73E+06 | 84.9 |
| 5 | 100 PE | Whole | ApW2 | 7.51E+06 | 6.30E+06 | 83.9 |
| 5 | 100 PE | Whole | ApW3 | 3.77E+06 | 3.23E+06 | 85.6 |
| 5 | 100 PE | Whole | AxenicW1 | 1.05E+07 | 8.91E+06 | 85.0 |
| 5 | 100 PE | Whole | AxenicW2 | 7.64E+06 | 6.45E+06 | 84.4 |
| 5 | 100 PE | Whole | AxenicW3 | 7.97E+06 | 6.71E+06 | 84.3 |
| 5 | 100 PE | Whole | ConvW1 | 2.17E+06 | 1.84E+06 | 84.6 |
| 5 | 100 PE | Whole | ConvW2^x^ | 1.04E+07 | 8.81E+06 | 85.1 |
| 5 | 100 PE | Whole | ConvW3 | 7.14E+06 | 6.06E+06 | 84.9 |
| 5 | 100 PE | Whole | LbrevW1 | 7.77E+06 | 6.69E+06 | 86.1 |
| 5 | 100 PE | Whole | LbrevW2 | 8.81E+06 | 7.60E+06 | 86.3 |
| 5 | 100 PE | Whole | LbrevW3 | 8.03E+06 | 6.88E+06 | 85.7 |
| 5 | 100 PE | Whole | LpW1 | 7.72E+06 | 6.55E+06 | 84.8 |
| 5 | 100 PE | Whole | LpW2 | 1.09E+07 | 9.40E+06 | 86.1 |
| 5 | 100 PE | Whole | LpW3 | 1.22E+07 | 1.05E+07 | 86.4 |
| 5 | 100 PE | Whole | YeastW1 | 1.07E+07 | 9.21E+06 | 86.0 |
| 5 | 100 PE | Whole | YeastW2 | 1.37E+07 | 1.18E+07 | 85.6 |
| 5 | 100 PE | Whole | YeastW3 | 1.37E+07 | 1.16E+07 | 84.8 |
| 6 | 150 PE | Gut | 3bac1 | 4.38E+06 | 2.39E+06 | 54.6 |
| 6 | 150 PE | Gut | 3bac3 | 5.02E+06 | 2.83E+06 | 56.4 |
| 6 | 150 PE | Whole | 3bacW1 | 9.11E+06 | 5.03E+06 | 55.2 |
| 6 | 150 PE | Whole | 3bacW2 | 6.13E+06 | 3.41E+06 | 55.6 |
| 6 | 150 PE | Whole | 3bacW3 | 2.04E+06 | 1.15E+06 | 56.3 |
| 6 | 150 PE | Gut | 4mic1 | 3.53E+06 | 1.97E+06 | 55.8 |
| 6 | 150 PE | Gut | 4mic2 | 4.70E+06 | 2.71E+06 | 57.7 |
| 6 | 150 PE | Gut | 4mic3^x^ | 3.78E+06 | 2.20E+06 | 58.1 |
| 6 | 150 PE | Whole | 4micW1 | 5.86E+06 | 3.21E+06 | 54.7 |
| 6 | 150 PE | Whole | 4micW2 | 6.97E+06 | 3.51E+06 | 50.4 |
| 6 | 150 PE | Whole | 4micW3 | 1.09E+07 | 5.90E+06 | 54.2 |
| 6 | 150 PE | Gut | Yeast4 | 2.80E+06 | 1.60E+06 | 57.0 |
| 6 | 150 PE | Gut | Yeast5 | 3.76E+06 | 2.08E+06 | 55.4 |
| 6 | 150 PE | Gut | Yeast6 | 3.68E+06 | 1.97E+06 | 53.5 |

*Value obtained from the “aligment_summary.txt” output following Tophat alignment of reads to v6.01 *D. melanogaster* genome.

^All libraries were sequenced on the Illumina HiSEq 2500 platform

x Aberrant behavior compared to rest of sample set, omitted from analysis.
